# Clinical Course, Radiological Manifestations, and Outcome of *Pneumocystis jirovecii* Pneumonia in HIV Patients and Renal Transplant Recipients

**DOI:** 10.1371/journal.pone.0164320

**Published:** 2016-11-08

**Authors:** Lukas Ebner, Laura N. Walti, Andri Rauch, Hansjakob Furrer, Alexia Cusini, Andreas M. J. Meyer, Stefan Weiler, Uyen Huynh-Do, Johannes Heverhagen, Spyridon Arampatzis, Andreas Christe

**Affiliations:** 1 Department of Diagnostic, Interventional and Pediatric Radiology, Inselspital, Bern University Hospital, University of Bern, Bern, Switzerland; 2 Department of Infectious Diseases, Inselspital, Bern University Hospital, University of Bern, Bern, Switzerland; 3 Department of Hypertension, Nephrology and Clinical Pharmacology, Inselspital, Bern University Hospital, University of Bern, Bern, Switzerland; University of Minnesota, UNITED STATES

## Abstract

**Background:**

*Pneumocystis jirovecii* pneumonia (PCP) is a frequent opportunistic infection in immunocompromised patients. In literature, presentation and outcome of PCP differs between patients with human immunodeficiency virus (HIV) infection and renal transplant recipients (RTRs).

**Methods:**

We conducted a cross-sectional study of patients with PCP based on the HIV and renal transplant registries at our institution. Radiological and clinical data from all confirmed PCP cases between 2005 and 2012 were compared.

**Results:**

Forty patients were included: 16 with HIV and 24 RTRs. Radiologically, HIV patients had significantly more areas of diffuse lung affection (81% HIV vs. 25% RTR; p = 0.02), more ground glass nodules 5–10 mm (69% vs. 4%; p = <0.001) and enlarged hilar lymph nodes were found only in HIV patients (44%). Cough and dyspnea were the most common clinical signs (>80%) in both groups. Duration from illness onset to hospital presentation was longer in the HIV patients (median of 18 vs. 10 days (p = 0.02)), implying a less fulminant clinical course. Sixty percent of PCP cases in RTRs occurred >12 months after transplantation. Lengths of hospitalization, admission rates to the intensive care unit, and requirements for mechanical ventilation were similar. Outcome in both groups was favourable.

**Conclusions:**

While important differences in radiological presentation of PCP between HIV patients and RTRs were found, clinical presentation was similar. PCP only rarely presented with fulminant respiratory symptoms requiring ICU admission, with similar results and outcomes for HIV patients and RTRs. Early diagnosis and treatment is mandatory for clinical success.

## Introduction

*Pneumocystis jirovecii* pneumonia (PCP) is one of the most frequent opportunistic infections [1,2] and remains one of the leading causes of morbidity and mortality in HIV patients and patients with impaired cell-mediated immunity [3].

In HIV patients, PCP typically manifests as an opportunistic infection mainly in patients with a CD4+ count of less than 200/μL. PCP is still one of the most frequent AIDS defining diseases in resource rich settings and its occurrence is a marker of inadequate access to care or poor adherence to drug therapy [4–6]. Overall, the incidence of PCP has decreased with widespread use of chemoprophylaxis and the early introduction of antiretroviral therapy in HIV patients. However, it remains a serious clinical problem due to the increasing use of cytotoxic and immunosuppressant therapies in special populations. Risk factors for PCP in patients without HIV infection are immunosuppressive treatment, e.g. glucocorticoids, and defects in cell-mediated immunity [7,8]. Approximately 15% of patients who undergo solid organ transplantation develop PCP in the absence of prophylaxis, especially in the early post-transplantation period [9,10]. In contrast to HIV patients, PCP in renal transplant recipients (RTRs) has often been associated with fulminant respiratory failure and adverse outcomes [11].

PCP may be suspected based on the patient’s history, clinical signs and symptoms, laboratory findings and abnormal imaging studies, although initial chest X-rays may have no pathological findings. A number of studies have been published on the different thoracic manifestations of PCP in chest computed tomography (CT) scans in HIV patients [12–14]. In general, the most pertinent finding is ground glass opacity (GGO) indicative of alveolar subtotal consolidation. Only few investigations have compared the clinical and radiological presentation of PCP in patient with distinct immunodeficiency in well-characterized groups, such as RTRs and HIV patients [9,15]

HIV-related PCP can progress rapidly and can be severe and lethal, but findings made by many working groups have shown that it progresses more slowly than PCP in other immunocompromised hosts. Early reports by Kovacs et al. [16] comparing the clinical manifestations of HIV-associated PCP to features of PCP in subjects with other immunosuppressive disorders identified more indolent disease in HIV patients. Both groups, however, had poor survival rates reaching almost 50% lethality. Later reports from Mansharamani and colleagues assessing management and outcome patterns in a large group of HIV and non-HIV patients highlighted the declining mortality rate in HIV-related PCP of less than 7% in contrast to a rate of 40% in non-HIV-infected PCP patients [11,16]. Recent studies have shown better survival of 86–92% in HIV patients than in other groups of immunocompromised patients. This may be due to increased PCP awareness and management in these patients during the past decades [17].

In order to explore the current characteristics and outcomes of PCP infections, we conducted a study based on two well-described immunosuppressed patient groups from our tertiary care university hospital: adults with HIV, and RTRs. The primary aim of this study was to compare CT manifestation patterns, current clinical features, treatment modalities and outcome in HIV patients and RTRs with confirmed PCP. Our hypothesis was that the different underlying immunosuppressive mechanisms are reflected in both the clinical and radiological manifestations and that outcomes of PCP differ in the two patient groups.

## Materials and Methods

The Bern University Hospital (Inselspital) is a tertiary-care urban teaching hospital in Switzerland treating >40,000 inpatients and more than 500,000 outpatient consultations per year. The current study was a cross-sectional analysis of confirmed cases of PCP, based on the local HIV (www.shcs.ch) and transplant recipients cohorts.

All transplantations were performed according to the criteria of the Swiss national transplant program based on the strict national laws and regulations for cell, tissue and organ donation (Swiss national law 810.21, https://www.admin.ch/opc/de/classified-compilation/20051806/index.html) and conform to the international standards. None of the transplant donors were coming from a vulnerable population and all (living) donors or next of kin provided written informed consent that was freely given. All research involving human participants have been approved by local the Cantonal Ethics of Berne (Kantonale Ethikkommission Bern). All clinical investigations have been conducted according to the principles expressed in the Declaration of Helsinki. Written Informed consent have been obtained from the registry participants. The local HIV and renal transplant registries in our institution were screened between 2005 and 2012 for patients with suspected PCP.

Inclusion was primary based on clinically and radiologically suspected PCP at admission plus one of the following criteria for further confirmation: 1) microbiological confirmation of Pneumocystis jirovecii by staining or PCR in sputum, bronchoalveolar lavage samples, or lung biopsies 2) successful antimicrobial PCP treatment. Successful treatment was defined as rapid clinical response, after initiation of the standard therapeutic regiment (trimethoprim-sulfamethoxazole) with accompanying resolution of typical radiological PCP findings during follow up radiological examinations. Follow-up radiological examinations (i.e. CT-Scans and Chest X-rays) were not systematically performed at predefined time points, since all the follow up examinations and treatment decisions were at the discretion of treating physicians. The image data was collected from the hospital PAC-system, anonymized and stored for further evaluation. After the randomization process, two radiologists with 4 and 15 years of experience in chest imaging performed a consensus read out of the cases. The readers were blinded to the clinical data, symptoms and results. The readers were not involved in the initial interpretation of the CT scans at the time of diagnosis.

### Patient demographics and clinical outcomes

Outpatient and inpatient medical records and laboratory and microbiological findings of all identified and confirmed cases in patients >18 years of age were retrospectively reviewed. Data were recorded on standardized forms, including age, gender, underlying medical conditions, HIV status and risk factors, peripheral CD4+ T-lymphocyte count, use of immunosuppressive agents before PCP diagnosis, and use of chemoprophylaxis against PCP.

### Image acquisition and interpretation

The radiologic images were acquired with 4-, 16-, 64- and 128-row scanners (all scanners were manufactured by Siemens, Forchheim, Germany) with a slice thickness of 1 to 2 mm. The cases were randomized before reading. The lung patterns were classified according to the Fleischner Society recommendations on the stratification of lung disease. In addition, the radiologists recorded the presence of pleural effusions and hilar and mediastinal lymphadenopathy (node > 1 cm).

### Statistical methods

For the statistical analysis, continuous data were compared using Student’s t test if normally distributed, or else using the Mann-Whitney test. Statistical analyses of non-continuous dichotomous data were compared using the chi-squared test or Fisher’s exact test, as appropriate. The absolute frequency of each lung pattern was recorded and compared between groups. In addition to the absolute pattern frequency, each patient was also analyzed individually. The standard error of frequency was assessed. All p-values were two-sided and the level of significance was p = <0.05. A post-hoc Bonferroni test was applied to evaluate significant differences between each assessed value. The statistical analysis was performed with the MedCalc Version 7.6.0.0 statistical software (MedCalc Software, Ostend, Belgium).

## Results

### Clinical findings

Our analysis included 40 patients with confirmed PCP: 24 RTRs and 16 HIV patients. Clinical and patient characteristics, treatment and PCP manifestation patterns, and outcomes in both groups are presented in Table 1. Both groups consisted mainly of male patients and the RTRs were significantly older than the HIV patients (p = 0.002). Unlike the HIV patients, due to the chronic kidney disease in RTRs, patients had multiple comorbidities such as hypertension and diabetes mellitus. Underlying pulmonary medical conditions, including chronic lung disease, were present to an equal extent in both groups. In 8/16 HIV patients PCP was the primary disease manifestation. In the remaining 8/16 HIV patients with previously established diagnosis 5 were ever under ART, but 4/5 patients were not under ART at PCP diagnosis (due to adherence problems (n = 3) and stop after primary infection (n = 1)). The patient under ART at PCP diagnosis was already for the last 17 years under treatment with changing adherence, viral load (23cp/ml) and CD4+ count (106 cells/μL) were low at PCP diagnosis. Concerning chemoprophylaxis, no RTRs were under prophylactic treatment at PCP diagnosis. PCP manifested in the majority of RTRs cases (19/24, 79%) more than 6 months after transplantation (range 0–144 month). Regarding chemoprophylaxis in HIV, in the 8/16 patients with previous diagnosed HIV, six had a CD4+ count below 200 cells/μL but only one was under prophylactic treatment (and only for the last 4 weeks, the other patients were not adherent to follow up visits and/or treatment), two patients had CD4+ counts of more than 200 cells/μL but with non-suppressed viral loads. Immunosuppressive agents prior to PCP diagnosis and median dose per day in RTRs at onset of PCP diagnosis are presented in Table 1. The standard immunosuppression regiment in RTRs consisted of a triple therapy with long term steroids at a median dose of 12.5mg, Mycophenolate mofetil and calcineurin inhibitors in the majority of the patients. Over half of the RTRs (14/24, 58%) had sought medical advice from our outpatient service in the last weeks before PCP diagnosis due to a suspected viral upper respiratory tract infection. The most common clinical PCP symptoms leading to hospital presentation, cough and dyspnea (>80% in both groups), had been present for a longer period than in the HIV patients (median 18 vs. 10 days in RTR, p = 0.02). There were fewer hospitalizations for PCP treatment in HIV patients. In contrast, all RTRs with PCP were hospitalized and most received adjunctive steroid therapy (22/24, 92%). Fever was present in >40% of patients in both groups. In 38/40 patients microbiological confirmation of *Pneumocystis jirovecii* was established by staining or PCR in sputum, bronchoalveolar lavage samples, or lung biopsies. In two RTRs, without microbial PCP confirmation, diagnosis was based upon typical clinical and biochemical constellation, comparison of radiological patterns CT-scans at disease manifestation with previous radiological findings, biochemical constellation and the clinical and radiological course of the disease after of PCP therapy initiation. The length of hospitalization, admission to the intensive care unit (ICU), and requirement for mechanical ventilation were similar between the groups (Table 1). Only one patient from the RTR group died during hospitalization due to pulmonary embolism. Three other patients died during follow up, two RTRs and one HIV Patient (all because of acute pulmonary infection other than PCP). Concerning the PCP treatment regimen used, 38/40 patients were treated with trimethoprim-sulfamethoxazole. One HIV patient with a mild allergic reaction to trimethoprim-sulfamethoxazole after therapy initiation and one RTR with a known trimethoprim-sulfamethoxazole allergy received atovaquone suspension as alternative treatment and secondary prophylaxis. Outcome of both patients was favorable. Kidney function in RTRs worsened during infection, but reached baseline values after PCP treatment completion. Secondary PCP prophylaxis was recommended to all patients.

**Table 1:** Demographics, clinical characteristics, and outcomes of PCP in renal transplant recipients vs. HIV-infected patients.

	RTR(n = 24)	HIV(n = 16)	
Characteristic			P-value
**Demographics**			
Age, median (range)	60(31–77)	44(31–66)	**0.002**
Male, n (%)	15/24(63)	11/16(69)	0.75
Follow-up years (range)	5.1(0–8.3)	4.5(0.7–9.7)	0.82
**Comorbidities**			
Chronic kidney disease, n (%)	24/24(100)	0/16(0)	**0.0001**
Hypertension, n (%)	21/24(88)	2/16(13)	**0.0001**
Heart insufficiency, n (%)	6/24(25)	1/16(6)	0.21
Diabetes mellitus, n (%)	9/24(38)	0/16(0)	0.03
Underlying pulmonary condition, n (%)	10/24(42)	6/16(38)	1.0
**Clinical symptoms**			
Before PCP diagnosis, days (range)	10(1–49)	18(1–77)	**0.02**
Fever, n (%)	9/20(45)	3/7(43)	1.0
Cough, n (%)	19/22(86)	9/10(90)	1.0
Dyspnea, n (%)	18/22(82)	8/10(80)	1.0
**Hospitalisation details**			
Admission to hospital, n (%)	24/24(100)	13/16(81)	**0.06**
Length of hospitalization, days (range)	11.5(3–75)	13(0–65)	0.8
ICU admission rate, n (%)	4/24(16)	1/16(6)	0.63
Need for mechanical ventilation, n (%)	3/24(13)	1/16(6)	0.63
**Group-specific characteristics**			
Months elapsed, median (range)			
from transplantation to PCP diagnosis	23(0–240)		
from HIV diagnosis to PCP		108(0–240)	
CD4^+^ count cl/μL median (IQR)		57(10–379)	
PCP as first manifestation of HIV, n (%)		8/16(50)	
ART naïve patients, n (%)		11/16(69)	
Under ART at PCP diagnosis, n (%)		1/16(6)	
**Immunosuppressive agents used in RTRs**			
Calcineurin inhibitors, n (%)	14/24(58)		
Cyclosporin, n (%)	10/24(42)		
Median dose/day, mg (range)	200(125–375)		
Tacrolimus, n (%)	4/24(17)		
Median dose/day, mg (range)	4.5(3–11)		
mTOR-Inhibitors, n (%)	6/24(25)		
Sirolimus, n (%)	2/24(8)		
Median dose/day, mg (range)	4.75(2.75–7)		
Everolimus, n (%)	4/24(17)		
Median dose/day, mg (range)	4.75(2.75–10)		
Antimetabolic agents			
Mycophenol mofetat, n (%)	18/24(75)		
Median dose/day, mg (range)	2000(750–2000)		
Prednison, n (%)	19/24(79)		
Median dose/day, mg (range)	12.5(5–50)		
**Prophylaxis**			
Under PCP-Prophylaxis at Diagnosis, n (%)	0/24 (0)	1/16(6)	
**Treatment regimen**			
Trimethoprim-sulfamethoxazole^1^, n (%)	23/24(96)	15/16(94)	
Duration, median days (IQR)	23(18–25)	21(11–34)	0.76
Receiving adjunctive steroid therapy, n (%)	22/24(92)	16/16(100)	0.51
**Outcome**			
30-day all-cause mortality, n (%)	1/24(4)	0/16(0)	
Died during follow-up, n (%)	2/24(8)	1/16(6)	1.0

Note: significant differences are given in bold font type; PCP = Pneumocystis jirovecii pneumonia, RTR = Renal transplant recipient, IQR = Interquartile range.

^1^ The two patients not treated with trimethoprim-sulfamethoxazole received atovaquone suspension due to known or apparent trimethoprim-sulfamethoxazole allergy.

### Radiological findings

The pattern frequency per lung segment is shown in Table 2 and the lung segment distribution of PCP in the RTR group is shown in S1 Table. The most frequent pattern was patchy ground glass opacity (GGO) in 92% of cases. The involvement of the lung was primarily multifocal (88%) with slight peripheral predominance (58%) and subpleural sparing (33%). In the HIV group, the most common pattern of PCP was patchy GGO (94%). The following patterns were identified in the chest CTs: nodules <5 mm (88%), reticulation (81%), mosaic GGO (75%) and nodules 5–10 mm (69%) (Table 2). Fifty percent of the patients had hilar or mediastinal lymphadenopathy. The peripheral regions of the lung were more affected (94%) than the perihilar regions (81%), with dominant areas of diffuse patterning (81%; S1 Table).

**Table 2:** Location and pattern frequency of PCP manifestations in renal transplant recipients and HIV-positive patients.

		RTR (n = 24)			HIV (n = 16)			Fisher's exact Test
		Prevalence/Pattern % frequency ± SD			Prevalence/Pattern % frequency ± SD			Bonferroni corrected p-value
**Location of disease**								
	Central lung affected	54.2	±	10.2	81.3	±	9.8	1.00
	Multifocal	87.5	±	6.8	81.3	±	9.8	1.00
	Peripheral	58.3	±	10.1	93.8	±	6.1	0.55
	Diffuse	25.0	±	8.8	81.3	±	9.8	**0.02**
	Subpleural sparing	33.3	±	9.6	50.0	±	12.5	1.00
	% Symmetric involvement of both lungs	60.8	±	23.8	65.9	±	28.1	1.00
**Patterns**								
Consolidation	All consolidations	20.8	±	8.3	31.3	±	12.5	1.00
	Bronchopneumogram present	12.5	±	6.8	18.8	±	9.8	1.00
Interstitial patterns	Reticulation	83.3	±	7.6	81.3	±	9.8	1.00
	Arcades	12.5	±	6.8	50.0	±	12.5	0.21
	Linear stripe	41.7	±	10.1	50.0	±	12.5	1.00
	Cysts	10.3	±	5.7	31.3	±	11.6	1.00
Ground glass patterns	Diffuse	16.7	±	7.6	56.3	±	12.4	0.31
	Patchy	91.7	±	5.6	93.8	±	6.1	1.00
	Mosaic	58.3	±	10.1	75.0	±	10.8	1.00
	Nodule <5 mm	66.7	±	9.6	87.5	±	8.3	1.00
	**Nodule 5–10 mm**	4.2	±	4.1	68.8	±	11.6	**0.0004**
Others	Pleural effusion	8.3	±	5.6	12.5	±	8.3	1.00
	Pneumothorax	4.2	±	4.1	6.3	±	6.1	1.00
	**Hilar lymphadenopathy**	0.0	±	0.0	43.8	±	12.4	**0.01**
	Mediastinal lymphadenopathy	16.7	±	7.6	50.0	±	12.5	1.00

Note: significant differences are given in bold font type; RTR = Renal transplant recipient, IQR = Interquartile range, SD = standard deviation.

**Fig 1:**
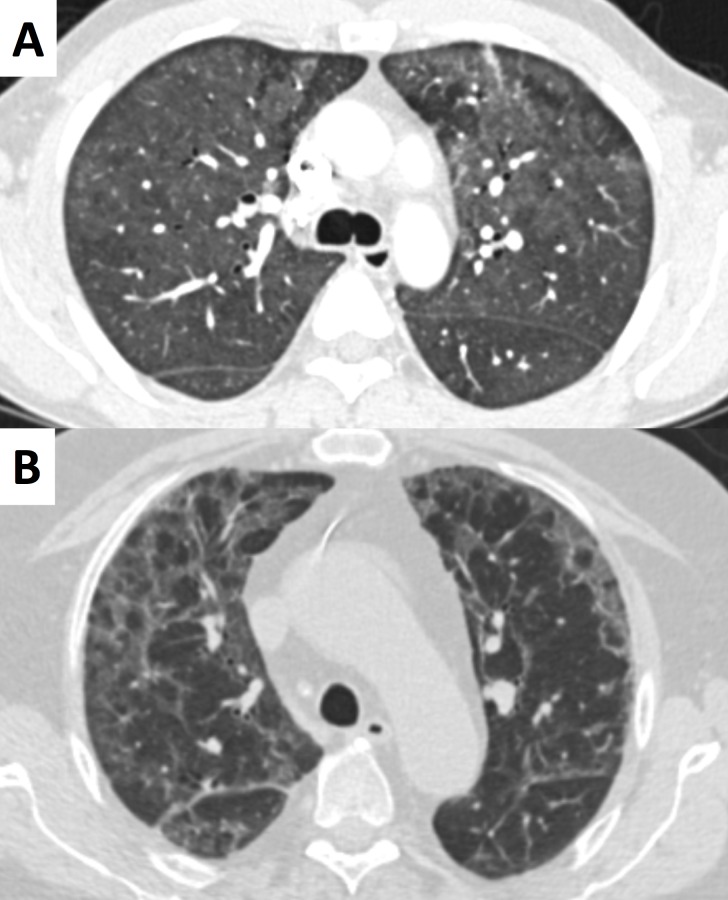
A) 45-year-old HIV-positive male patient with PCP with typical diffuse ground glass opacities in both lungs. B) 58-year-old male patient 5 years after kidney transplantation who developed PCP with multifocal patchy ground glass opacities and reticulation.

**Fig 2:**
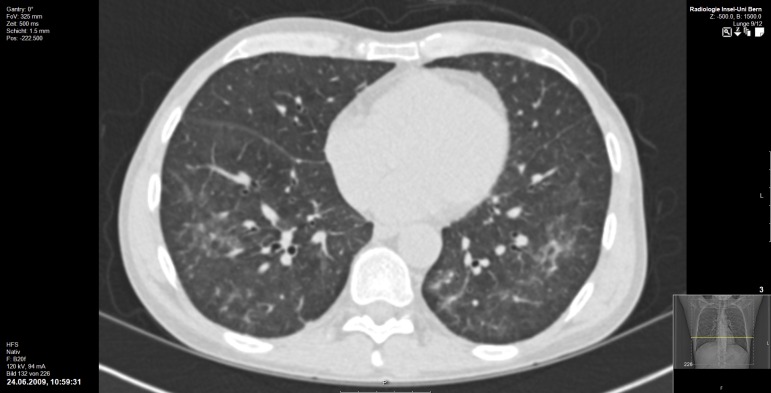
HIV-positive individual (49-year-old, male), CDC stage C3 with PCP. Faint, peripheral ground glass opacities with diffuse distribution in the upper and lower lobes in both lungs. This finding was significantly more frequent in HIV-positive individuals. Subpleural sparing is also present.

**Fig 3:**
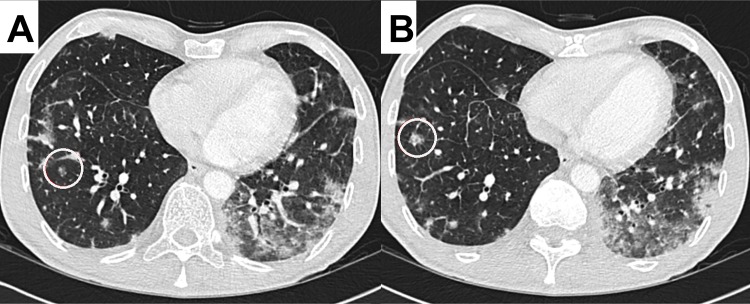
55-year-old HIV positive male patient with PCP presenting with ground glass nodules in a diffuse distribution, predominantly in the right lower lobe (circles in image A and B). This finding was significantly more prevalent in the HIV-positive study population. Patchy opacifications are also evident in the left lower lobe.

**Fig 4:**
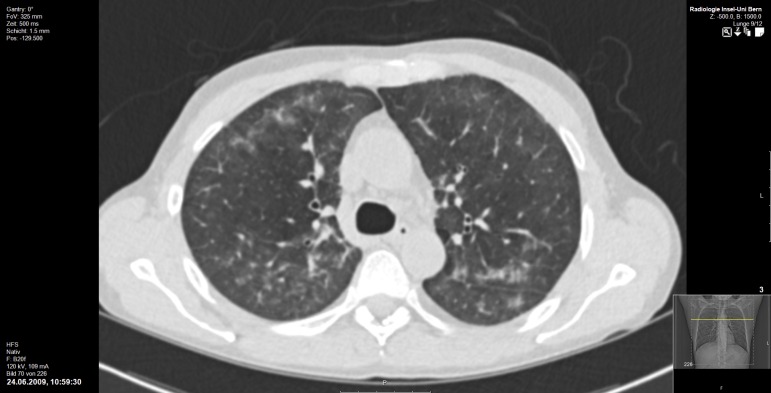
70-year old, male RTR presenting with PCP. Bilateral, patchy consolidations with areas of ground glass attenuation. Septal thickening in the outer portions of both lungs is present. Also, bilateral, peribronchial cuffing can be seen. Note the absence of hilar lymphadenopathy.

HIV-associated PCP infection had a significantly more diffuse pattern in 81% of patients (seen in only 31% in the RTR group; p = 0.036). Ground glass nodules (GGNs) were also significantly more frequently of intermediate size (5–10 mm) in 69% of HIV patients compared with only 4% in the RTR group (p < 0.0001). Hilar lymphadenopathy was identified in the HIV group (44%) but not the RTR group (p = 0.01). Lung segments 1 and 5 were less affected in the RTRs (S1 Table). The RTR and HIV groups showed patchy GGO in 92% and 94% of patients, reticulation in 83% and 81%, GGN <5 mm in 67% and 88%, GGN 5–10 mm in 4% and 68.8 ± 11.6%, mosaic GGO in 58% and 75%, and mediastinal lymphadenopathy in 50% (Table 2). Distinct PCP manifestation patterns in HIV patients and RTRs are presented in Figs 1–4. Radiological follow-up was not performed systematically since the follow up examinations and treatment decisions were at the discretion of treating physicians.

## Discussion

The primary objectives of the present study were to compare radiological manifestations, clinical course, treatment and outcome of PCP between HIV patients and renal transplant recipients (RTRs). Similarities and important differences were found between the two groups, both clinically and radiologically. With regard to distinct radiological patterns, enlarged hilar lymph nodes were found only in HIV patients with PCP. Also, a significantly more diffuse pattern of scattering and larger GGNs were present in HIV patients.

Our radiological findings are in agreement with previous studies that have examined the patterns in the lung associated with PCP in patients with and without HIV [3,18–20]. PCP shows a predilection for the upper lobes; also, subpleural sparing and asymmetric distribution of GGO were present in both of our groups. By further separating GGO into diffuse and patchy consolidations, we were able to demonstrate that a diffuse GGO pattern was predominantly present in the HIV patients. PCP affected a greater lung volume in HIV patients than RTRs and displayed a wider variety of patterns, although cystic lesions have been identified more often in HIV patients. Interestingly, this radiological pattern is now being seen less frequently [3].

A further radiological pattern seen much more often in our HIV patients was the high frequency of GGNs with a diameter of 5–10 mm. These sub-solid nodules were very rare in our RTRs, which may reflect partial granulomatous inflammation with subtotal alveolar consolidation as a response to PCP [14]. Enlarged hilar lymph nodes occurred only in our HIV patients, which may suggest that the lymph node may be associated with the HIV as the primary infection and not with the PCP [13]. The absence of pneumothorax may reflect the timely detection and appropriate treatment of the disease, thus reducing PCP-associated complications.

Regarding the clinical course, the period between onset of symptoms and diagnosis in RTR was significantly shorter than in HIV patients. Although we confirm that the course of PCP in HIV patients is often that of a gradually progressive disease with distinct clinical and radiographic manifestation patterns different from those in RTR, our findings are not in agreement with those of numerous published studies that report different outcomes for PCP in HIV patients and otherwise immunosuppressed patients, with unfavorable outcomes in the latter [14,16,21]. The clinical course in our groups of patients was similar, with prolonged symptoms present before diagnosis, although of longer duration and less marked in our HIV patients. Most RTRs had respiratory symptoms before PCP was diagnosed. This initial clinical characteristic may reflect a less acute course for PCP in RTRs or a triggering upper respiratory co-infection, as reported by earlier studies [9,21,22].

Nearly two-thirds of the RTRs developed PCP more than one year after transplantation, at a time when routine PCP prophylaxis is no longer being given. The durations of hospitalization, rates of acute respiratory failure, and need for mechanical ventilation were similar in our HIV patients and RTRs, and we also observed favourable outcomes in RTRs.

Also, almost all of our RTRs received adjunctive steroid therapy after PCP diagnosis. However, it remains unclear whether the intermittent higher steroid dose (prophylactic stress dose) given to the RTRs because of the suspected prodromal respiratory co-infection before PCP became manifest might increase the risk of developing PCP or contribute to the favorable outcome. We plan subsequent studies to explore the transmission dynamics and the influence of intensified immunosuppression (e.g. due to antirejection therapies) in early- vs. late-onset PCP in our RTRs.

Our findings have important clinical implications for caregivers of renal transplant and HIV-infected patients. First, in contrast to prior reports, the clinical course of PCP seems to be more subtle and is now rarely life-threatening in both in HIV and non-HIV patients, since only few of our patients had to be admitted to ICU and needed mechanical ventilation. Despite the overall favorable outcome in our patients, RTRs should be observed carefully, and since PCP may present with a late onset and initial chest X-ray findings may be unspecific, further radiological procedures including CT imaging appear to be mandatory in transplant and immunocompromised patients [12,23]. Second, our findings might reflect the changes in clinical PCP awareness, time of diagnosis and more rapid treatment in high-risk populations, resulting in decreased morbidity and mortality due to PCP. The importance of appropriate and timely PCP prophylaxis must also be emphasized, since improvements in implementing preventive measures can further decrease hospital admissions and adverse outcomes [24].

Our study has several limitations. Foremost, the relatively small numbers of patients and single center design limits the generalizability of our results. Rates of PCP among solid organ transplant recipients were reported to be lowest for RTR and highest among lung and heart-lung transplant patients. Therefore, our results from RTR cannot be generalized to other patient groups. Also in all but two patients microbiological confirmation of PCP was provided by staining or PCR in sputum, bronchoalveolar lavage samples, or lung biopsies. In the remaining two RTRs without definite microbial confirmation, PCP diagnosis was based upon typical clinical and biochemical constellation, as well as radiological initial manifestation. These two patients might have suffered from other parenchymal lung disease, but the clinical presentation characteristic and radiological patterns were similar to other RTRs. Furthermore, we did not fully address current patient lifestyles and the exact adherence to drug therapy, both factors with a major impact on PCP manifestation and outcome. Additional studies that include large, prospectively selected groups of PCP-infected immunocompromised patients should address these shortcomings.

In conclusion, our radiological data suggest that PCP is associated with a distinct lung pattern in HIV patients and RTRs. PCP only rarely presents nowadays with fulminant respiratory symptoms requiring ICU admission, and this is also the case in heavily immunocompromised patients such as RTRs. Early diagnosis and treatment is mandatory for clinical success.

## Supporting Information

S1 Table Lung segment involvement in PCP in HIV-positive and renal-transplant recipients. (DOCX)

## Abbreviations

CT: computed tomography
HIV: human immunodeficiency virus
GGO: ground glass opacity
GGN: ground glass nodule
PCP: *Pneumocystis jirovecii* pneumonia
RTR: renal transplant recipient
