# Supplementary material for: Clinical Course, Radiological Manifestations, and Outcome of Pneumocystis jirovecii Pneumonia in HIV Patients and Renal Transplant Recipients
Source: PLoS One. 2016 Nov 8;11(11):e0164320. doi: 10.1371/journal.pone.0164320 (PMC5100884; doi:10.1371/journal.pone.0164320)
Supplement: S1 Table — (DOCX) [file pone.0164320.s001.docx]

**S1 Table.** **Lung segment involvement in cases of pneumocystis pneumonia in HIV-positive and renal-transplant recipients.**

| **Right lung** | **HIV-positive patients** | **RTR** |  | **Left lung** | **HIV-positive patients** | **RTR** |
| --- | --- | --- | --- | --- | --- | --- |
| **Segments** | *Involvement (%)* | *Involvement (%)* |  | **Segments** | *Involvement (%)* | *Involvement (%)* |
| **LS 1** | 100 | 89.7 |  | **LS 1** | 87.5 | 75.9 |
| **LS 2** | 100 | 86.2 |  | **LS 2** | 87.5 | 93.1 |
| **LS 3** | 100 | 96.6 |  | **LS 3** | 87.5 | 82.8 |
| **LS 4** | 81.3 | 82.2 |  | **LS 4** | 87.5 | 86.2 |
| **LS 5** | 87.5 | 82.8 |  | **LS 5** | 87.5 | 69 |
| **LS 6** | 93.8 | 82.8 |  | **LS 6** | 93.8 | 89.7 |
| **LS 7** | 93.8 | 75.9 |  | **LS 7** | 100 | 89.7 |
| **LS 8** | 93.8 | 86.2 |  | **LS 8** | 93.8 | 96.6 |
| **LS 9** | 100 | 93.1 |  | **LS 9** | 100 | 89.7 |
| **LS 10** | 100 | 96.6 |  | **LS 10** | 100 | 89.7 |

Note: LS = lung segment, modified lung segments of the lung. RTR = renal transplant recipient
